# Mini Review: Synergizing Driven Quantum Dynamics, AI, and Quantum Computing for Next-Gen Materials Science

**DOI:** 10.1021/acs.jpclett.5c02390

**Published:** 2025-11-05

**Authors:** Opeyemi S. Akanbi, Jack P. Shannon, Jerome Delhommelle, Caroline Desgranges

**Affiliations:** † Department of Physics & Applied Physics, 14710 University of Massachusetts Lowell, Lowell, Massachusetts 01854, United States; ‡ Department of Chemistry, 14710 University of Massachusetts Lowell, Lowell, Massachusetts 01854, United States

## Abstract

The design of next-gen materials has undergone remarkable progress in recent years, as evidenced by the emergence of automated platforms combining artificial intelligence (AI)-driven synthesis planning and robotics for execution. In this Mini-Review, we analyze how synergistic approaches that combine driven quantum dynamics, AI/machine learning, and quantum computing accelerate the discovery and design process of quantum materials with enhanced properties and novel functionalities. Building on the capabilities of each of the three methods, synergistic approaches can provide access to the materials’ response to time-dependent fields, enable the rapid exploration of vast design spaces, and identify novel quantum phases and materials with optimal properties. We examine recent successes in next-gen materials science for quantum batteries, colloidal quantum dots solar cells, quantum phototransistors, rare-earth-free materials, and applications in quantum information processing. We conclude with a discussion of recent research efforts in AI-for-quantum computing and quantum machine learning for next-gen materials discovery.

## Graphical abstract

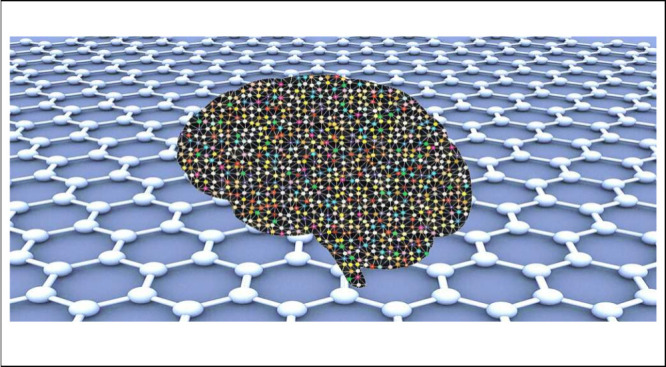

Next-generation (next-gen) materials are central to many emerging technologies, driving progress in computing, energy, communications, sustainability, and biomedical applications. They can be defined as innovative materials that exhibit markedly improved performance or novel functionalities that conventional materials do not have. Next-gen materials with enhanced properties include, for example, low-dimensional materials, such as graphene^1^ and transition metal dichalcogenides.^2^ Such materials have high carrier mobility, mechanical flexibility, and tunable band gaps that make them especially suited to the development of flexible electronics, high-speed transistors, and quantum devices. Examples of novel functionalities include self-healing^3^ or the ability to respond to environmental stimuli.^4,5^ The applications of next-gen materials are wide-ranging^6^ and include, among others, phase-change materials in computing,^7^ metamaterials and topological photonic structures for communication systems,^8^ perovskites^9^ as efficient alternatives to silicon, organic photovoltaics as flexible and transparent energy harvesting materials,^10^ solid-state electrolytes and high-capacity electrode materials for next-gen lithium-ion and sodium-ion batteries,^11^ as well as materials instrumental to environmental sustainability such as photocatalysts for water splitting^12^ and metal–organic frameworks (MOFs) for carbon capture.^13,14^ In short, next-gen materials are at the forefront of materials science, where the convergence of multidisciplinary innovation and sustainability is driving technological progress.

There are, however, several outstanding challenges that need to be addressed before next-gen materials science can fully deliver on its promises. The identification and synthesis of next-gen materials hinge on the efficient as well as economical exploration of an extremely large design space. This has become crucial in recent years as materials scientists have increasingly focused on designing next-gen hybrid materials. The design of such hybrid materials involves integrating two or more components from distinct chemical, structural, or functional classes, i.e., organic and inorganic units,^13^ metals and semiconductors,^15^ or biological and synthetic frameworks. The components are then interfaced or covalently linked to create new materials with emergent properties and novel functionalities. This strategy has enabled remarkable advances, but has also made the design, control, and optimization of the properties of next-gen materials increasingly complex. In addition, several global challenges remain, including the dependence on rare-earth elements and the growing need for circular economy materials that are durable, recyclable, modular, and energy-efficient.

To address these formidable challenges, researchers are increasingly exploring solutions that combine traditional scientific domains, Artificial Intelligence (AI), and Machine Learning (ML), as well as robotic platforms. Such platforms are poised to enable the autonomous discovery of next-gen materials. Recent work has shown, for example, that the combination of AI-driven chemical synthesis planning and a robotically controlled experimental platform was capable of synthesizing several drugs and drug-like compounds.^16^ Computer-aided synthesis planning was first performed, using a neural network model that was trained on data contained in the scientific literature and predicted synthetic routes with a high probability of success. After adding implementation details provided by expert chemists in the form of recipe files, the experiments were carried out by a modular continuous-flow platform that was automatically reconfigured by a robotic arm. Work is currently underway to generalize these approaches to design quantum materials and fabricate quantum devices. A recent study focused, for example, on the atomic-scale manufacturing of carbon-based quantum materials with single-bond precision, which is of key significance to the development of next-gen spintronics and, more broadly, to quantum information technologies. Su et al.^17^ introduced the concept of a chemist-intuited atomic robotic probe that integrated probe chemistry knowledge and AI to enable atomically precise single-molecule manipulation and obtain single-molecule quantum π-magnets with single-bond precision. Quantum AI algorithms are expected to further enhance the computational screening of thousands of candidate materials exhibiting properties such as superconductivity, magnetoresistance, or specific catalytic activity, and program robots to adapt experimental parameters in real time for optimal synthesis. The resulting closed-loop platforms, in which quantum-AI-driven robotics autonomously discover and obtain new materials for quantum computing, energy storage, and next-generation electronics, are set to revolutionize materials science.

In this Review, we build on these developments and show how recent synergistic efforts that leverage driven quantum dynamics, Machine Learning (ML), and Quantum Computing (QC) have enabled breakthroughs in the design of next-gen quantum materials. Each of these three pillars provides a distinctive capability in the next-gen quantum materials design process. Driven quantum dynamics provides an in-depth understanding of systems with correlated electrons, superconductivity, and topological phases, and provides insight into the ultrafast nonlinear processes that underpin emergent functionalities in quantum materials. ML is accelerating next-gen materials discovery by enabling rapid predictions of properties, phase diagrams, and synthesis pathways from high-throughput data sets^18^ and, as a result, the efficient exploration of the extremely large design space. QC offers a revolutionary platform for solving complex many-body problems beyond classical capability, both in its analog form, with the development of a multipurpose ultracold atoms-based platform^19^ where changes in the arrangement and interactions can enable the observation of exotic quantum states, and in its digital form, with the development of hybrid classical-quantum algorithms for quantum materials science.^20^ We discuss how these three interconnected pillars together define a roadmap for advancing next-gen quantum materials science and provide an integrated framework that will considerably advance next-gen quantum materials science in the coming years (see Figure 1). We then examine examples of synergistic approaches that combine driven quantum dynamics, ML, and QC have led to the discovery of next-gen quantum materials, before finally drawing the main conclusions from this work.

**Figure 1:**
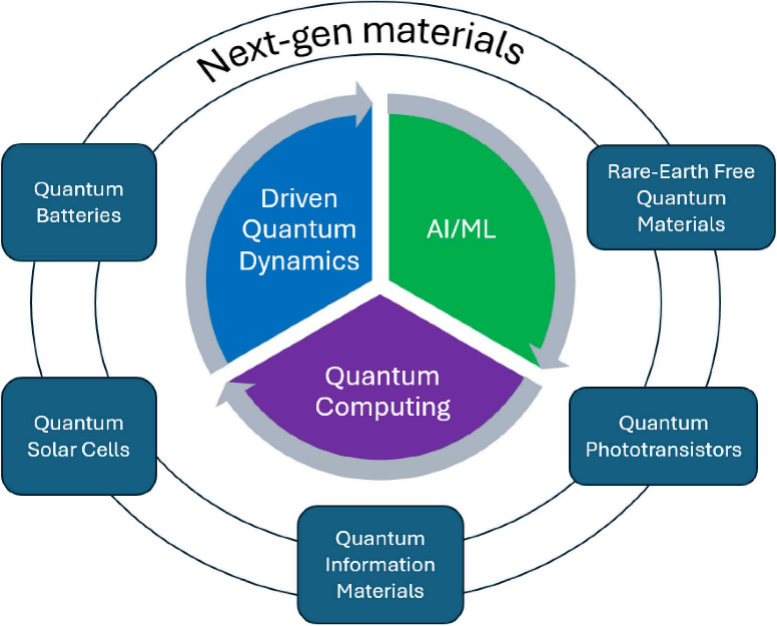
Schematic diagram highlighting how synergistic approaches that combine driven quantum dynamics, ML, and QC accelerate the design of next-gen quantum materials. Through theory, experiments, and computations, driven quantum dynamics provides insight into the ultrafast nonlinear processes that underpin emergent functionalities in quantum materials. The generated data can then be used to train ML/AI models that enable the efficient exploration of the extremely large design space and the rapid prediction of properties, phase diagrams, and synthesis pathways. The ML predictions can then be tested either experimentally using analog quantum computers or computationally using digital quantum simulations and inform the next round of design changes to improve the functionalities of next-gen materials.

Driven quantum dynamics describes quantum physical systems subjected to a time-dependent external field or periodic driving.^21^ A generic Hamiltonian for driven quantum dynamics can be written as

$$Ĥ\left(t\right)={Ĥ}_{0}+\mathrm{V̂}\left(t\right)$$ 1

where *Ĥ*_0_ denotes the time-independent part and *V̂*(*t*) the time-dependent drive. Time-dependent Hamiltonians often generate novel phenomena that are not accessible with stationary quantum mechanics. Hänggi and Grifoni^22^ showed that the interplay between the drive parameters and the intrinsic properties of the system impacted quantum tunneling, population dynamics, and transport processes. If we now consider a two-level quantum system (TLS) subjected to a laser pulse with a frequency close to the transition frequency between the two levels, we obtain the well-known Rabi oscillations, with a Hamiltonian given by

$$Ĥ\left(t\right)=\frac{\hbar \omega_{0}}{2}{\mathrm{\sigma ̂}}_{z}+\hbar \Omega \;\cos\left(\omega t\right){\mathrm{\sigma ̂}}_{x}$$ 2

where *σ̂*_*z*_ and *σ̂*_*x*_ denote Pauli matrices, ω_0_ the TLS transition frequency, ω the laser frequency, and Ω the Rabi frequency. The coherence resulting from this coupling can be leveraged to obtain a superposition of the two quantum states and thus arbitrary quantum states and qubits in quantum computers. TLS is thus a building block for realistic systems, that include multiple reservoirs (interactions) or complex laser fields. The TLS-laser interaction is a paradigmatic example of how external drives trigger quantum transitions, manipulate quantum states, and lead to novel quantum technologies.

In experiments, ultrafast lasers with pulses ranging from 10^–12^-10^–15^ s can drive quantum systems out of equilibrium and engineer advanced properties. They can trigger a photoinduced insulator-to-metal transition in VO_2_ ^23^ (see Figure 2), and create metastable, or ”hidden” states, enabling light-induced superconductivity in copper oxides.^24^ They can also help unravel complex properties such as magnetic dynamics in quantum materials. Shining X-ray light on Sr_2_IrO_4_ results in a loss in long-range magnetic order and in the formation of photocarriers that induce strong and nonthermal magnetic correlations,^25^ thereby opening the door to light-controlled magnetism. With the advent of attosecond (10^–18^ s)-resolved technology, it is now possible to observe and control electronic processes in materials with very high precision, allowing direct insight into ultrafast electronic dynamics that was previously inaccessible. This suggests new strategies to engineer materials into a given quantum state by controlling quantum correlations and creating quantum phenomena on demand.^26^

**Figure 2:**
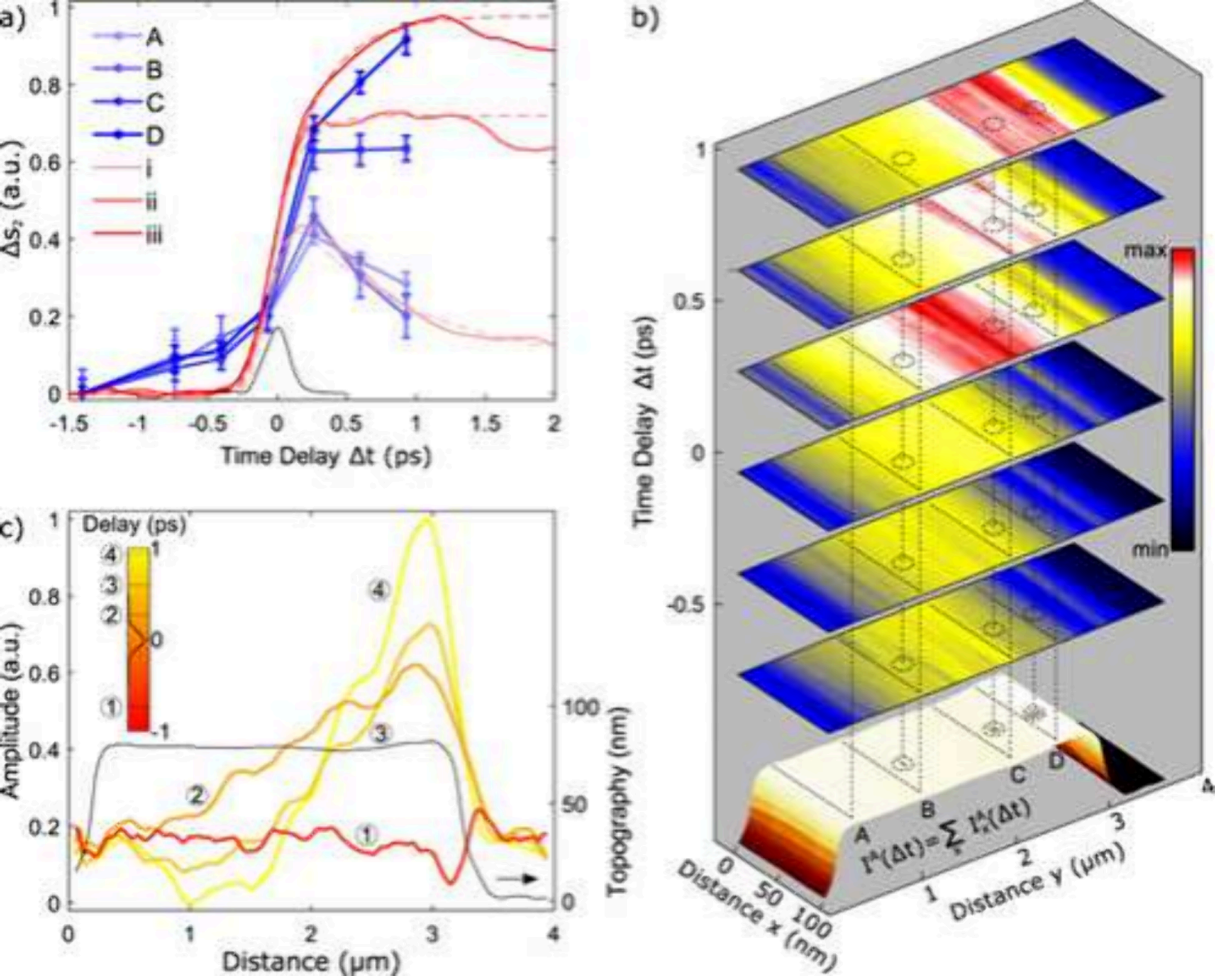
Ultrafast nanoimaging of the photoinduced insulator-to-metal (IMT) transition in VO_2_. (a) Near-field pump–probe time traces at different points on a microcrystal (in red in the bottom plot), with full time traces obtained from a series of spatiotemporal images (in blue). (b) Line profiles for several pump–probe time delays across the microcrystal. (c) Images showing spatial and temporal variations during IMT. Adapted with permission from Nano Lett. 2016, 16, 3029–3035 27096877 10.1021/acs.nanolett.5b05313. Copyright 2016 American Chemical Society.

Quantum simulation can be performed either experimentally (analog simulator) or with a quantum algorithm (digital simulator). Analog quantum simulations use controllable quantum systems to mimic complex quantum behavior. Ultracold atom-based quantum simulators^27^ model quantum magnetism in iron or nickel by using Rydberg atoms. Similarly, ultracold atoms in optical lattices, i.e., periodic potential landscapes created by the interference of counter-propagating laser beams that form crystal-like arrays of light, can simulate topological insulators^28^ and mimic their electronic band structures, allowing the study of edge states, quantum Hall effects, and other topological phenomena. Trapped ion quantum simulators^29^ leverage long-range Coulomb interactions to engineer various spin models and lattice gauge theories. Recently, So et al.^30^ used multispecies trapped-ion crystals to model electron transfer between donor and acceptor states by controlling the donor–acceptor gap, electronic and vibronic couplings, and bath relaxation dynamics. By measuring the response of the ion states to laser pulses, the system simulates how molecules absorb and emit light, providing insight into the underlying quantum dynamics of electron transfer.

The principles discussed above have led to the design of, e.g., ultrafast switches that respond almost instantly to light pulses or electric fields. Hybrid quasiparticles known as exciton-polaritons can be created by incorporating quantum wells in a tunable optical microcavity.^31^ Exciton-polaritons exhibit novel quantum states at relatively high temperatures. They can form quantum condensates and propagate over long distances, which makes them ideal for next-generation computing, telecommunications, and quantum information processing. Nanoplasma switches can also be obtained by shining a laser pulse on a nanoparticle. The resulting nanoplasma then expands and recombines on ultrafast time scales, enabling ultrafast electrical switching.^32^ Since driven quantum systems are controlled with light or electric fields rather than temperature, they provide highly energy-efficient solutions for quantum memory elements and quantum sensors. Applying ultrafast pulses to manipulate quantum states allows information to be written, read, and erased both quickly and safely. High-resolution quantum sensing of electro-magnetic fields can also be achieved with single-molecule quantum sensors, composed of iron atoms and a PTCDA molecule attached to the tip of a scanning tunneling microscope,^33^ and has applications in electronics, photonics, and medical imaging. Although advances in driven quantum dynamics have shed light on the complex properties of next-gen quantum materials, they also suffer from several limitations as driven quantum dynamics is often unable to efficiently explore vast design spaces. Furthermore, traditional approaches are often unable to capture hidden correlations and emergent phenomena in nonequilibrium systems. However, these limitations can be addressed by integrating artificial intelligence (AI) and machine learning (ML) with driven quantum dynamics to identify patterns in data, reduce computational bottlenecks, and enable predictive modeling for accelerated discovery in driven quantum systems.

High-throughput computational screening has become essential in quantum materials discovery and design. ML models can process millions of material properties and automatically detect patterns or correlations.^34^ Once ML models are trained on large DFT data sets, they can predict band gap, conductivity, or magnetic ordering for a wide range of materials, including superconductors, spin liquids, and topological insulators.^35^ For example, recent work on 2D layered materials^36^ focused on TMDs (Transition Metal Dichalcogenides), a class of materials with applications in nanoelectronics and optoelectronics. TMDs have a chemical formula of MX_2_, where M is a transition metal atom (such as Mo, W, or Ti) and X is a chalcogen atom (such as S, Se, or Te) and a structure consisting in a single layer of metal atoms sandwiched between two layers of chalcogen atoms, forming thin sheets that are only a few angstroms wide. Starting from a DFT data set of 10^5^ material candidates and using intercalation energy as a screening criterion to tune the electronic and optical properties, Kastuar et al.^36^ were able to identify ∼ 50 promising hybrid quantum materials from the data set. ML models can also leverage explainable AI (XAI) techniques to make predictions interpretable and transferable.^37^ Traditional ML models, and especially the widely used deep neural networks, tend to act as ”black boxes”. This means that they provide accurate predictions for the properties of materials without any insight of how design choices, or input features, impact the material properties. On the other hand, XAI methods such as SHAP (SHapley Additive exPlanations) values can measure the contribution of each feature to the model’s predictions and thus identify which design choices are crucial to the performance of the material.^38,39^

ML can also be used to refine our understanding of dynamics in quantum systems.^40^ Δ-ML models^41–44^ increase the accuracy of high-throughput DFT calculations by learning the difference (or correction) between low-level (higher efficiency and lower accuracy) quantum calculations, e.g., DFT calculations using approximations such as the generalized gradient approximation (GGA) or semilocal functionals that often lead to systematic errors in properties like band gaps, and high-level (lower efficiency and higher accuracy) quantum calculations, e.g., many-body perturbation theory approaches and hybrid functionals. By training a Δ-ML model on a data set of differences between the two levels of theory for selected materials, the Δ-ML model will be able to predict high-level corrections for new systems. This means that high-accuracy calculations will no longer be needed since low-level quantum calculations, augmented with the Δ-ML corrections, will provide access to highly accurate band gaps, magnetic properties, and topological invariants. In a recent study of Cd-based chalcogenides,^41^ Δ-learning was used to learn the difference between transition levels calculated using DFT with the PBE functional (low-level DFT) and with the HSE06 functional (high-level DFT). Adding the Δ-ML correction then led to predictions within ∼ 0.21 eV (RMSE) for the test data set. As a result, this type of approach enables high-throughput screening and accelerated discovery of quantum materials.^45^ Similarly, Δ-ML models can improve the efficiency of QM/MM (Quantum Mechanical/Molecular Mechanical) calculations, in which the system is divided into an active region, where quantum effects are prevalent and reactions take place, treated at the computationally expensive QM level, and the rest of the system, treated at the classical (MM) level.^46^ Recent work has revealed that a Δ-ML model can be trained to learn the difference between a high-accuracy *ab initio* QM/MM calculations and low-accuracy semiempirical QM/MM calculations.^47^ Applying the Δ-ML correction to a different semiempirical QM/MM calculations thus enables obtaining a high-accuracy result at a modest computational cost. Moreover, ML models known as convolutional neural networks (CNNs) can be trained on quantum many-body simulation data to predict magnetic phase diagrams, identify finite-temperature phases in strongly correlated Fermion systems, and even predict the system behavior upon doping.^48^ The ability of AI techniques to interpolate and, to some extent, extrapolate from quantum simulation data considerably reduces the computational cost of quantum calculations.

Data-driven approaches efficiently explore large chemical and structural spaces to identify promising quantum materials. Combining data-driven models, autonomous experimentation, and predictive analytics enables the design and manufacturing of new materials for quantum technologies and functional materials. The CAMEO (Closed-Loop Autonomous Materials Exploration and Optimization)^49^ platform uses real-time experimental analysis with AI-driven decision-making. By autonomously analyzing data from combinatorial libraries and synchrotron diffraction, this approach can rapidly map phase diagrams and optimize compositions, as shown recently for thin films of the phase-change memory material GeSbTe. GNoME (Graph Networks for Materials Exploration)^50^ recently identified 2.2 million stable inorganic crystal structures from the Materials Project database, including 381,000 new materials that were more stable than any previously known combinations. GNoME also trained highly accurate machine-learned interatomic potentials for molecular dynamics simulations, facilitating the discovery of material candidates for batteries, photovoltaics, and microchips. Finally, the recent development of quantum-enhanced machine learning,^51^ which combines classical data processing with quantum algorithms, is expected to further accelerate materials discovery.

Physics-guided machine learning (PGML) uses physical and chemical laws to improve model accuracy, interpretability, and generalization. PGML reduces prediction errors by constraining the solution space to physically plausible regimes and embedding domain knowledge such as symmetry, conservation laws, and known reaction pathways.^45,52^ Physics-guided generative models^52^ have been applied to design new crystal structures by embedding crystallographic rules, such as symmetry and space groups. The predicted ∼ 500 structures were then confirmed as stable materials using DFT, establishing the benefit of using physics-based models. PGML models also perform well for quantum materials discovery. Such models have the ability to generalize to new materials and facilitate materials optimization by rapidly identifying highly significant features.^45^ A PGML approach was recently used to design new shape memory alloys (SMAs)^53^ by combining elemental descriptors, engineered using scientific principles, with features derived from heat treatment processes and preprocessed with thermodynamic and kinetic models. The model enabled reliable predictions of new SMA compositions and processing routes, leading to a highly efficient exploration of the high-dimensional composition-process-property space. Furthermore, physics-informed machine learning interatomic potential (MLIP) frameworks have been developed, leveraging symmetry, equivariance, and many-body interactions explicitly. Examples include MACE (Molecular Atomic Cluster Expansion),^54^ which uses high-order atomic cluster expansions and equivariant message-passing networks, as well as FLARE (Fast Learning of Atomistic Rare Events),^55^ based on Gaussian process regression with active learning, and NequIP,^56^ an E(3)-equivariant graph neural network architecture. By embedding physical symmetries and constraints, these frameworks ensure physically consistent predictions and better transferability across diverse chemical environments and accelerate materials discovery by enabling accurate and computationally efficient simulations. Although AI and ML provide powerful approaches for uncovering patterns and accelerating materials discovery, their effectiveness can be limited by the exponential increase in complexity of quantum many-body systems, particularly for strongly correlated and time-dependent systems. These challenges highlight the need for quantum computing (QC), which offers fundamentally new ways to represent and process quantum states that are beyond the reach of classical computing. By bridging AI and ML with QC, we can address problems that are otherwise intractable, opening the door to the predictive and scalable modeling of next-gen quantum materials and devices.

Simulating quantum systems on classical computers is computationally intensive since the number of possible quantum states grows exponentially with system size. This is due to quantum superposition, where the state of a system is described as a combination of many possible configurations. From a mathematical standpoint, quantum states are represented as vectors in a high-dimensional space called Hilbert space that describes all possible states of a quantum system. As the system size increases, the dimension of this space grows exponentially, making classical simulation challenging. To address this challenge, Kane proposed building a quantum computer based on the nuclear spins of phosphorus atoms embedded in a silicon chip.^57^ The information was encoded onto the nuclear spins, logical operations on individual spins were performed with electric fields, spin measurements were carried out using currents of spin-polarized electrons, and metal gates were used for control. This opened the door to analog and digital quantum simulators. When a quantum device serves as an analog quantum simulator, the interactions in the simulator are engineered to mimic the Hamiltonian of a quantum material. Using a quantum device based on strongly interacting nuclear spins attached to a diamond surface,^58^ Cai et al. were able to map out phase diagrams and identify new superconducting phases. In a digital quantum simulator,^59^ a quantum system is mapped onto qubits, or information units, so that each qubit represents a quantum degree of freedom (quantum state of a spin). The quantum algorithm is then programmed with quantum gates and circuits to mimic the interactions between particles.^20,60^

Simulation-driven discovery uses analog and digital quantum simulations to model and predict the properties of quantum materials (see Figure 3 for an analog simulation of a two-qubit system). An analog simulation was recently performed on a multipurpose ultracold atoms-based platform.^19^ Changing the arrangement and interactions between atoms provided access to the response of superconducting and magnetic quantum materials, thereby enabling the observation of exotic quantum states, such as superfluids. On the digital side, near-term quantum hardware is increasingly efficient in simulating realistic materials such as metals, semiconductors, or complex chemicals.^61^ This is achieved by devising strategies that reduce the number of steps (circuit depth), by performing efficient mappings from electrons to qubits, and by using compilers that tailor the quantum circuit to a given material. This has led to computational speed-ups of several orders of magnitude. Digital­(step-by-step)-analog­(real-time) simulations can also allow for an analysis of how electrons interact in materials. Using reconfigurable qubit architectures, the connections between qubits, and thus the interactions between electrons, can be engineered to mimic target materials, thereby shedding light on catalysis and magnetism in 2D materials.^62^

**Figure 3:**
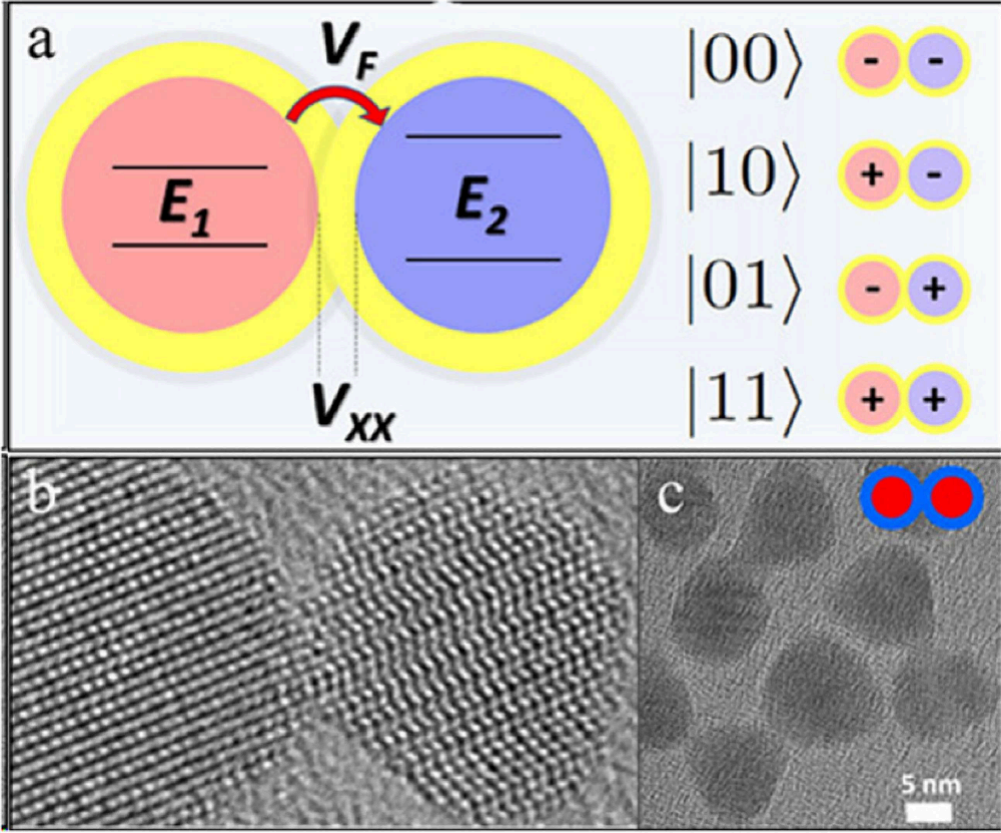
(a) Two-qubit system based on the excitonic states of coupled semiconductor nanocrystals (NCs). E_1_ and E_2_ denote the exciton energies in each nanocrystal (*E*_1_ < *E*_2_ and Δ*E* = *E*_2_ – *E*_1_), V_F_ the Förster transition dipole–dipole interaction strength, and V_*xx*_ the biexciton static dipole–dipole interaction strengths. The four possible states are characterized by having an exciton present (1) or absent (0) on each NC. (b) and (c) Transmission electron microscopy (TEM) images of CdSe/CdS core/shell nanocrystals (heterodimers).^63^ Reprinted with permission from ref 63. Copyright 2021 American Chemical Society.

Quantum simulations can also model charge and energy transfer processes. Using a digital quantum simulator and a quantum algorithm that propagates the time-evolution of a vibronic Hamiltonian, Motlagh et al.^64^ used a digital quantum simulator and a quantum algorithm to propagate the time-evolution of a vibronic Hamiltonian and model exciton transport and charge transfer in anthracene dimers and at anthracene-fullerene interfaces. This showed that quantum algorithms could efficiently simulate complex nonadiabatic vibronic dynamics in realistic systems that are beyond reach for classical methods and thus accelerate the design of novel organic solar cell materials. Such models can guide the design of more efficient solar cells. An analog quantum simulation of vibronic dynamics was performed on a mixed-qudit-boson (MQB) approach by mapping molecular vibrational modes to bosonic degrees of freedom (phonons in trapped ions) and electronic states to qudit states. The simulator was programmed to mimic a target molecule and successfully predicted the vibronic spectrum for SO_2_.^65^ Analog quantum simulators can also model exciton dynamics by tailoring spin-phonon interactions and including ”reservoir engineering” to account for controlled dissipation.^66^ Qubits, quantum gates, and quantum circuits can capture the complex time evolution of excitons.^67^ This recent study showed the significance of non-Markovian interactions with the environment on exciton dynamics and demonstrated the onset of ”memory-assisted” quantum transport.

Quantum algorithms are enabling increasingly accurate simulations of complex materials. Such algorithms include the variational quantum eigensolver (VQE)^68^ and quantum phase estimation (QPE).^69^ VQE is a hybrid quantum-classical algorithm that combines short coherent quantum evolutions with classical optimization. VQE is suitable for near-term quantum hardware and yields accurate ground-state energies for molecules and quantum magnets on photonic, superconducting, and trapped ions qubits.^70^ QPE determines the eigenvalues of a Hamiltonian, but requires long coherence times and low error rates, a challenge for current noisy intermediate-scale quantum (NISQ) devices. To overcome this, innovative techniques such as iterative QPE, low-depth algorithms, and hybrid approaches combining QPE with classical postprocessing and ML have been developed. Quantum machine learning (QML) algorithms are a promising tool for quantum materials discovery. Quantum support vector machines can perform classification tasks on perovskites and superconductors.^9^ Quantum active learning can help optimize the structure of doped 3Al*@*Si_11_ nanoparticles and outperform classical active learning,^71^ while quantum neural networks^72^ can capture complex relationships in data sets and predict a wide range of material properties. QML is thus poised to significantly impact next-gen materials design.

We now highlight synergy stories in which driven dynamics, ML, and QC are integrated into complete workflows. These examples demonstrate how the three pillars jointly accelerate the design of quantum batteries, next-gen photovoltaic materials, rare-earth-free magnets, and topological materials for quantum information applications.

Quantum batteries are emerging as next-gen energy storage systems because of their fast charging speed and high energy density. Unlike conventional electrochemical batteries, which rely on ion transport and redox chemistry, quantum batteries take advantage of quantum coherence, entanglement, and superabsorption to accelerate charging while preserving high extractable energy. In pioneering work,^73^ Quach et al. showed that filling an organic semiconductor microcavity with molecular dyes enabled superabsorption under short laser pulses, marking the first experimental step toward practical quantum batteries. From a theoretical standpoint, the Dicke quantum battery model, where *N* two-level systems are collectively coupled to a photonic cavity, demonstrates how entanglement and cooperative quantum phenomena can greatly enhance the charging power of quantum batteries.^74,75^ Recent advances have integrated machine learning approaches such as reinforcement learning to optimize the charging process by dynamically modulating the cavity detuning and coupling strengths. This resulted in an increased extractable energy, or ergotropy, reduced quantum fluctuations, i.e., improved charging precision, while maintaining cooperative speedup of the charging time during almost the entire charging cycle.^76^ Together, these advances highlight that synergistic approaches that combine many-body quantum physics, artificial intelligence, and photonic platforms are driving progress in the field of quantum batteries and make these materials as compelling solutions for next-gen energy storage.

Colloidal quantum dot (CQD) solar cells have emerged as next-gen photovoltaic materials thanks to their tunable band gaps and compatibility with flexible and low-cost substrates. CQD solar cells^77^ can be designed by mixing CQDs with an ink that contains ligands and keeps CQDs dispersed.^78^ The dispersion can then be printed and coated on surfaces, making manufacturing straightforward. Through size changes, CQDs can also be tailored to absorb specific parts of the solar spectrum, including infrared light, and, as such, are highly promising for next-gen photovoltaics. Tandem, also known as double junction, CQD solar cells and multiple junction CQDs solar cells^77^ involve stacking multiple CQD layers to absorb a wide range of solar wavelengths and maximize efficiency. Combining data-driven methods with experimental approaches can accelerate the discovery of optimal synthesis pathways. Machine Learning and Bayesian optimization can be used to model the parameter space for a synthesis and help optimize the synthesis to obtain a product with specific properties. This approach was recently applied to determine that using a growth-blocking agent, together with a high Pb:S ratio, a low injection temperature, and adding metal chlorides were instrumental to obtaining smaller and monodisperse PbS CQDs.^79^ The approach consists in a feedback loop between the ML model and the synthesis, which starts with the model providing predictions for parameter combinations that achieve the synthetic goals, followed by data collection during experiments based on these predictions, which are then fed back to the model to allow for a gradual improvement in the accuracy of the predictions and a refinement of the parameters of the synthesis. Building on this paradigm, recent efforts have focused on coupling AI with experiments to design laboratory automation frameworks and develop closed-loop platforms for CQD materials discovery.^80^ Such platforms will enable the rapid identification and validation of the processing conditions leading to enhanced device performance and accelerate the translation of CQD science into scalable next-gen solar power solutions.

Quantum phenomena such as exciton dynamics, quantum coherence, and topological effects are instrumental in the design of next-gen optoelectronic devices. Polaritonic quantum devices (see Figure 4), such as quantum phototransistors, leverage hybrid light-matter quasiparticles known as polaritons formed as a coherent superposition between an electromagnetic field (photons) and an optically active polarization (exciton) in semiconductor or organic microcavities.^82^ These devices offer promising pathways for ultrafast, energy-efficient photonic switching and quantum information processing.^83^ Quantum correlation in polaritonic systems has recently been observed in low polariton excitation regime, which suggests that these systems could be used in quantum computing. This has prompted the development of deterministic quantum logic gates governed by polariton-polariton two-body interactions through a carefully chosen arrangement of integrated circuits of propagating single polaritons.^84^ This opens the door to applications of polaritons in photonic quantum computation and in quantum metrology. Finally, it has also been suggested that this strong light-matter coupling could also be used to control chemical reactivity either by inhibiting or catalyzing chemical reactions at room temperature. Recent work has focused on developing Machine Learning models to predict the impact of the vibrational strong coupling on chemical reactivity for a series of experimentally relevant molecules.^85^ Using the neuroevolution potential (NEP) framework, Schäfer et al. were able to train an artificial neural network, comprising a single hidden layer, with DFT data for the potential energy surface. The molecular structure was represented by a set of spatial descriptors, i.e., functions of interatomic distances that included interactions up to 4-body interactions. The resulting neuropotential was then used to compute forces and propagate the equations of motion for a reactive system, leading to the observation of a frequency-dependent rate constant, that is characteristic of polaritonic chemistry, and of changes in enthalpy and entropy in agreement with the experiment. This approach marked a significant step toward the AI control and optimization of polaritonic quantum systems and their nonlinear dynamics.

**Figure 4:**
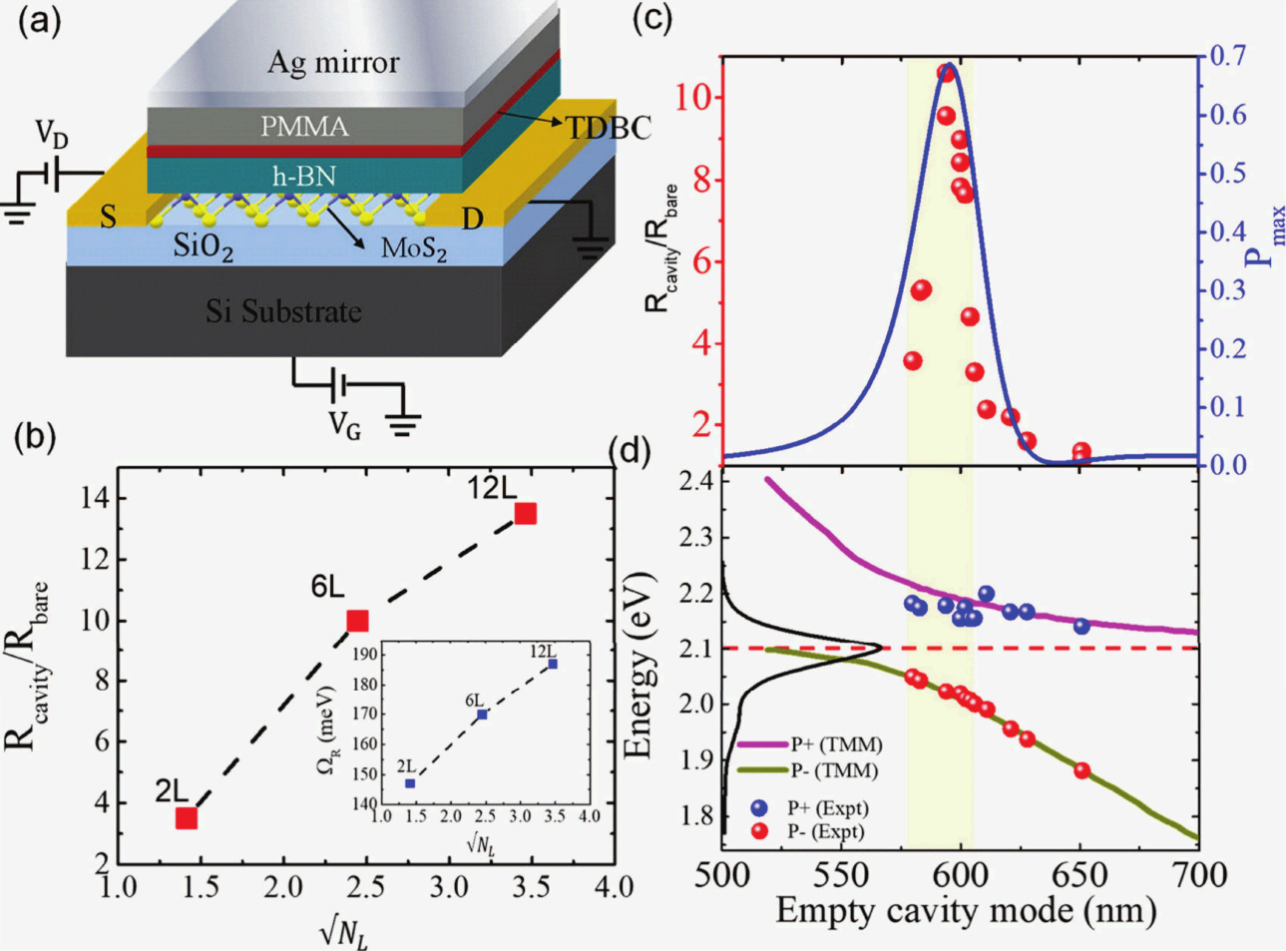
Polaritonic quantum device: cavity-electrical output of a quantum phototransistor:^81^ (a) Metal-oxide-semiconductor field-effect transistor Fabry-Pérot cavity containing a donor–acceptor pair. (b) Photoresponsivity ratio against $\sqrt{N_{L}}$ , where *N*_*L*_ denotes the number of layers of TDBC (inset: Rabi splitting). (c) Photoresponsivity ratio (in red) at 590 nm laser excitation wavelength plotted against the empty cavity mode position and calculated probability (in blue) of energy transfer. (d) Dispersion data for different phototransistors by tuning the cavity mode position (P+ and P- polaritonic states). Transfer matrix method (TMM) data are shown as pink and green, while the black curve corresponds to the absorbance of the bare TDBC thin film. Reprinted with permission from ref 81. Copyright 2024 American Chemical Society.

Finding substitute quantum materials for critical and rare-earth materials is crucial to applications in electronics, energy, manufacturing, and quantum computing. For instance, rare-earth elements such as Neodymium (Nd) are often added to permanent magnets in electric vehicles and wind turbines. In superconducting qubits, circuits use Niobium (Nb) to form Josephson junctions (a weak link between two superconductors that enables tunneling of Cooper pairs, which is a key building block for superconducting qubits), which are essential to the control of quantum states. Quantum and data-centric approaches can help identify sustainable substitutes. High-throughput quantum mechanical calculations can guide nanoscale synthesis on how to engineer quantum defects in materials.^86^ In the WS_2_ material, replacing S with Co creates a quantum defect with promising properties for applications in computing, telecommunications, and sensors. The ”Quantum Defect Genome” database gathers quantum defect properties for a wide range of host materials to facilitate the global search for critical material substitutes. Rare-earth-free (REF) materials^87^ can also be discovered by combining high-throughput quantum calculations, data mining, and experimental synthesis. Starting from the Materials Project database, Sakurai et al.^88^ used an adaptive genetic algorithm to discover new REF magnetic materials (see Figure 5) whose properties were evaluated by DFT calculations. Specifically, they uncovered several new Fe-, Co-, and Mn-rich magnetic compounds that exhibited significant magnetic anisotropy, large magnetization, and high Curie temperature, making them especially promising for data storage applications.

**Figure 5:**
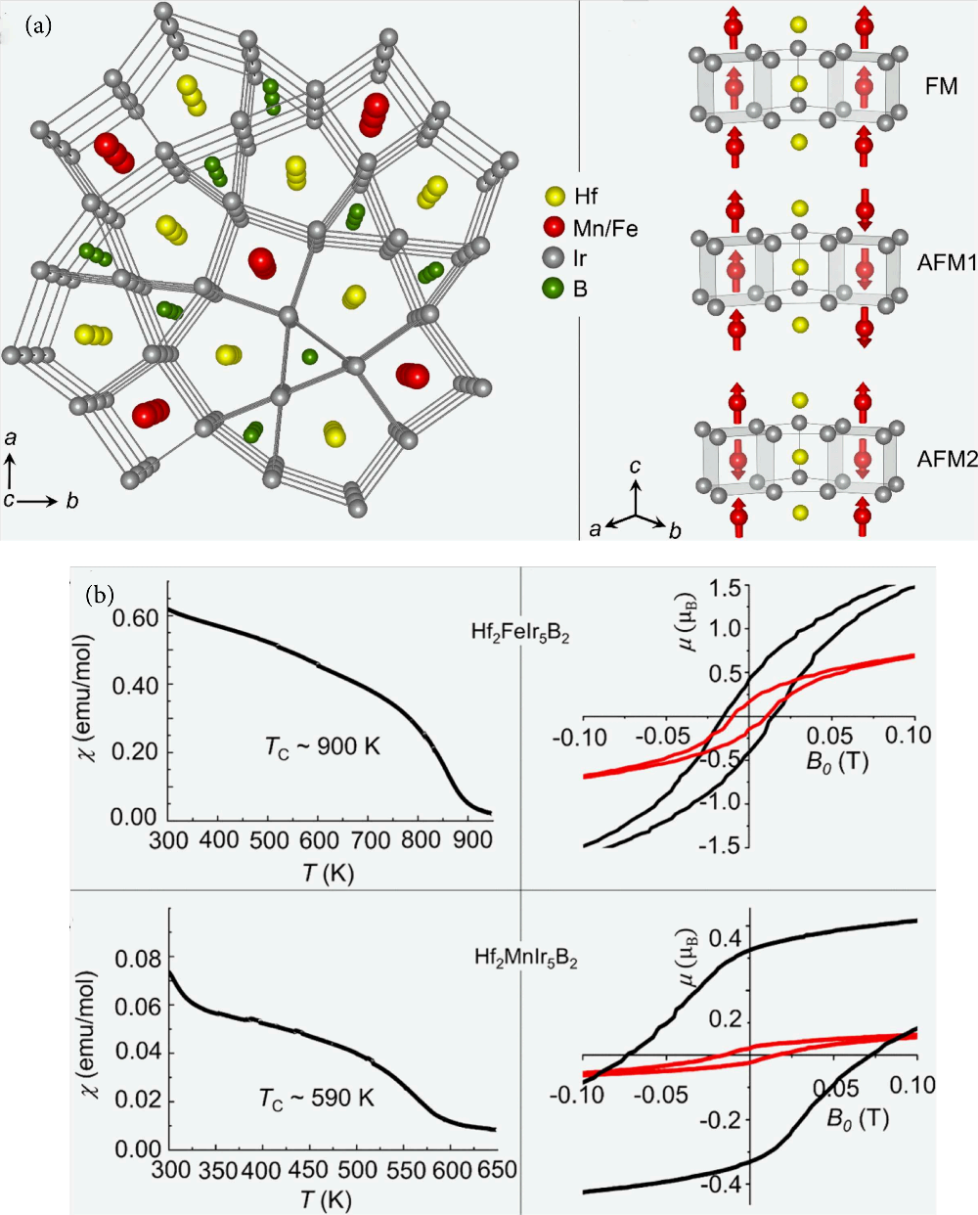
Rare-earth free magnets. (a) Crystal structure of Hf_2_MIr_5_B_2_ (M = Fe, Mn) viewed along [001] (left) with different models of magnetic chains (right). (b) Magnetic susceptibility measured at 1 T for Hf_2_FeIr_5_B_2_ (top left) and Hf_2_MnIr_5_B_2_ (bottom left), with an hysteresis loop measured at 5 K (black) and 300 K (red) for Hf_2_FeIr_5_B_2_ (top right) and Hf_2_MnIr_5_B_2_ (bottom right).^89^ Reprinted with permission from ref 89. Copyright 2021 American Chemical Society.

Topological and emergent quantum phenomena are crucial to applications in next-gen electronics and quantum computing. Topological transitions were observed about 20 years ago when Bernevig et al. confined a HgTe quantum well layer of variable width between two CdTe barrier layers and reported the onset of the quantum spin Hall effect and a topological phase transition.^90^ These materials give rise to unexpected phenomena, acting as insulators in their interior, as conductors of electricity on their surface, and exhibiting surface conduction states that are highly resistant to disorder and imperfections.^91^ Topological phenomena play an important role in quantum computing. For example, Majorana zero modes^92^ are defined as particle-like excitations that form at boundaries or defects in topological superconductors. Majorana Zero Modes (MZMs) are also protected by the material topology and are highly resistant to noise and errors. This property is crucial to the development of reliable quantum computing. From an experimental standpoint, MZMs can be realized using Majorana nanowires, which are hybrid structures of superconductors and semiconductors. A major challenge that has limited the search for MZMs in solid-state platforms is the presence of random disorder in experimental samples.^93^ ML models, specifically convolutional neural networks trained on conductance plots, were recently developed to determine the disorder landscape of Majorana nanowires.^94^ Similarly, ML models can also help address challenges associated with the use of MZMs in quantum computing. Recent work has shown how a ML approach known as the covariance matrix adaptation evolution strategy algorithm could be leveraged to automate the tuning of gate arrays.^95^ This ML-aided strategy allowed to fully recover Majorana zero modes that were destroyed by disorder in a case study of Majorana wires with strong disorder. Topological states can also be observed in Moiré heterostructures. Such structures can be obtained by stacking two or more ultrathin atomic layers (graphene or TMDs) with a slight twist or lattice mismatch between them. This misalignment creates a repeating interference pattern, called a Moiré superlattice, that can trap electrons and result in the emergence of strongly correlated quantum phases.^96^ AI-based approaches can accelerate the design of layered 2D materials with precisely controlled electronic properties by suggesting the selection of constituent layers, stacking sequences, and relative orientations. Recent work showed how AI-guided automated workflows could plan, run, and analyze electronic structure calculations for model 1D Moiré structures.^97^ The results obtained on the 1D models were shown to apply to realistic 2D systems and inform how Moiré band structures with target electronic properties could be obtained, thereby accelerating the computational design of Moiré superlattices. In addition, AI can also help address outstanding challenges in theoretical studies of Moiré supperlattices due to the strong correlation effects and the large size of Moiré unit cells. To this end, a neural network-based wave function methodology was recently developed by training the neural network wave function using a variational quantum Monte Carlo algorithm.^98^ This ML-based approach showed that a sequence of emergent Wigner phases form in TMD materials over a wide range of particle fillings. The AI-augmented computational design of quantum materials and the AI-guided exploration of their quantum phases is expected to drive progress in the emerging field of topologically protected quantum computing.

ML, quantum algorithms, and Floquet engineering are increasingly used to guide the design of new quantum materials, leading to more efficient design protocols. ML workflows increasingly rely on automated processes that leverage multiple ML models, as recently shown in the computationally guided design of REF magnetic materials.^99^ The aforementioned study used a crystal graph convolutional neural network trained on DFT data to achieve a high-throughput screening of material candidates and an adaptive genetic algorithm to suggest new material candidates. Quantum algorithms are also enabling the simulation of realistic materials on near-term quantum computers.^61^ Using compact Hamiltonian representations significantly reduces the number of qubits and circuit depth required and allows for the study of strongly correlated materials, such as SrVO_3_, on currently available hardware. Advanced quantum embedding techniques are now capable of accurately modeling strongly correlated systems, as shown in a recent VQE-in-DFT study of triple bond breaking in butyronitrile on a quantum computer.^100^ Finally, Floquet engineering has emerged as a powerful design strategy for quantum materials via the application of periodic external drives, such as intense laser fields or microwave pulses. Subjecting materials to these time-dependent perturbations reveals new quantum phases and phenomena that only exist out of equilibrium, including Floquet topological states, ultrafast spin dynamics, and nonequilibrium excitonic effects. Recent experiments have demonstrated how exotic virtual quantum states could be observed in monolayer TMDs with ultrafast laser pulses,^101^ opening new avenues for ultrafast spintronics and next-gen quantum devices.

In this Mini-Review, we showed how recent advances have led to the discovery of next-gen quantum materials with enhanced performance, energy efficiency and sustainability, and capable of addressing the demands of novel technologies such as quantum information processing and quantum computing. Our analysis revealed that the design of next-gen quantum materials required new paradigms and synergistic approaches that combined driven quantum dynamics, AI, and quantum computing. Using theory, computations, and experiments, driven quantum dynamics shows how external fields uncover ”hidden” quantum states and novel phenomena, leading to the design of nanoplasma switches, with ultrafast electrical switching, or superconducting quantum materials. By training AI models on quantum materials data sets, we can optimize the composition of materials to achieve a specific property and identify promising material candidates with generative AI methods. Recent examples of ML-guided designs include predictions of how defects trigger new quantum phases and ML models showing how the intercalation of ions in 2D materials improve their properties. Recent advances in quantum precision have also enabled the design of analog quantum simulations, in which an experimental setup models a quantum system and sheds light on its behavior. Ultracold atoms in optical lattices can, for example, mimic the band structure of topological materials. Furthermore, after harnessing superposition, entanglement, and interference, quantum materials can become hardware and enable quantum information processing. Quantum algorithms, as well as hybrid algorithms that mix classical and quantum approaches, are now capable of accelerating quantum mechanical calculations on currently available quantum hardware. Significant progress in quantum ML is also expected over the next few years. We anticipate that the combination of quantum calculations, quantum ML, and quantum computing will enable the rapid exploration of phase diagrams and provide accurate property predictions, which are both key to the design of next-gen materials. More generally, the integration of AI with quantum computing is set to significantly impact the discovery process. In AI-for-quantum computing approaches, ML and reinforcement learning (RL) can help manage the impact of noise on quantum platforms^102^ and make quantum processors more reliable and efficient, with RL’s ability to predict efficient quantum circuit designs and optimized gate sequences.^103^ Neural networks and Bayesian optimization can automate the tuning and characterization of quantum devices.^104^ Neural networks rapidly infer optimal parameters, while Bayesian optimization guides the experimental search by balancing exploration and exploitation, reducing the number of experiments needed. This approach minimizes manual intervention and accelerates convergence to optimal settings, resulting in improved coherence times and faster operation speeds.^104^ Moreover, quantum computers are especially well suited to handle large data sets and complex optimization problems. As a result, quantum algorithms can speed up the training of ML models and data processing, e.g., as preprocessing units for classical AI inference tasks in electronic structure computations. Beyond quantum materials, generative AI and quantum computing are expected to become a transformative research tool with the *de novo* generation of molecules for next-gen materials given recent successes in the drug discovery field.^105^
